# Hemorrhagic Stroke Due to Varicella Zoster Virus Vasculopathy

**DOI:** 10.7759/cureus.36604

**Published:** 2023-03-23

**Authors:** Reese Hofstrand, Rafael Portela, Ryan Juneau, Chika Okafor, Ryan Watts

**Affiliations:** 1 Internal Medicine, Cape Fear Valley Medical Center, Fayetteville, USA; 2 Cardiothoracic Surgery, Campbell University School of Osteopathic Medicine, Lillington, USA

**Keywords:** hiv, mri brain, vasculitis, punctate hemorrhage, varicella-zoster

## Abstract

Varicella-zoster virus (VZV) vasculopathy is a granulomatous vasculitis that has a wide variety of clinical presentations. It is most common in patients with HIV not on anti-retroviral therapy (ART) with low cluster of differentiation (CD)4 cell counts. This disease affects the central nervous system and can cause small intracranial bleeds. Our patient presented with stroke-like symptoms in the setting of recent VZV reactivation in the ophthalmic distribution with HIV on ART. Her MRI scan showed a small punctate bleed and the CSF workup was consistent with VZV vasculitis. She was treated with 14 days of acyclovir and five days of high-dose steroids with clinical improvement to baseline.

## Introduction

Varicella-zoster virus (VZV) has been known to cause chickenpox and in some cases resurface as shingles. Shingles occur due to the reactivation of the virus within the ganglion of a dorsal root, cranial nerve, or autonomic nervous system with spread to the corresponding dermatome or neural tissue. This typically starts with pain, tingling, or itching preceding the development of the unilateral rash. Shingles can be associated with complications including post-herpetic neuralgia, ophthalmologic disease, motor dysfunction (such as Bell’s palsy), transverse myelitis, and vasculitis [1,2].

Varicella-zoster virus vasculopathy, which has previously been called granulomatous angiitis, post-varicella arteriopathy, and varicella zoster vasculitis, is a granulomatous vasculitis that has a wide variety of clinical presentations. This occurs due to the reactivation of the virus in a cranial nerve ganglion followed by the spread and infection of the blood vessels. It is characterized by vessel wall damage, usually transmural inflammation, with multinucleated giant cells and with or without epithelioid macrophages. Central nervous system (CNS) small or medium vasculopathy is most common in immunocompromised individuals, including those with HIV infection and organ transplant recipients. However, HIV vasculitis is uncommon and is not typically at the top of the differential for patients presenting with acute neurological symptoms. There are lumbar puncture studies that will suggest the diagnosis in patients in whom the presence of VZV is suspected. Heterogenous presentations can occur considering that VZV can also precipitate arterial dissection, intracranial aneurysms, and venous sinus thrombosis [3]. Among HIV-infected individuals not receiving antiretroviral therapy (ART), CNS infection was detected at autopsy in 1.5% to 4.4% of deceased patients and had documented vasculopathy and leukoencephalitis. This was most prevalent in patients with severe cluster of differentiation CD4 cell depletion.

## Case presentation

A 70-year-old African-American female with a past medical history significant for chronic kidney disease (CKD) IV, coronary artery disease status post-myocardial infarction, HIV (~33 years, compliant with ART), and distant history of seizures presented to the ED via ambulance for altered mental status. History was obtained from a family member who states the patient was watching television when she "blanked out and stopped responding." The patient was unable to recall this event but later reported that two to three weeks prior to admission she was diagnosed with shingles in the left ophthalmic distribution with a persistent burning sensation in the same area. She did not receive treatment for shingles at that time.

**Figure 1:**
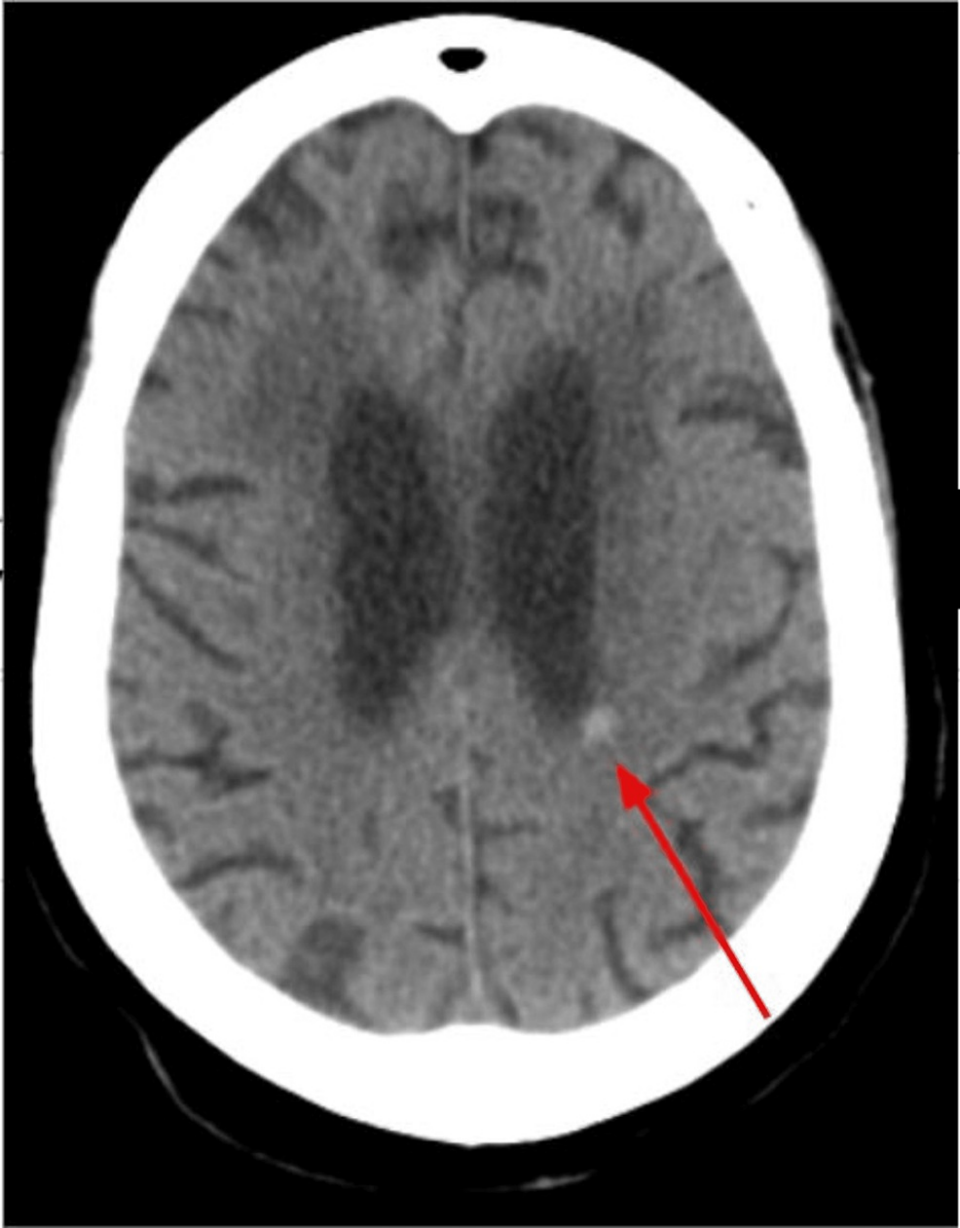
CT head without contrast showing small hyper-dense focus at the left centrum semiovale, suggestive of a small hyper-dense mass or subacute hemorrhage

When she presented to the ED, she had a left-sided facial droop which improved back to baseline prior to the initial stroke exam performed in the ED. Her initial exam showed no focal extremity weakness, no cranial nerve deficits, normal speech pattern, no sensation deficits, and no coordination deficits. Initial vitals were temperature 36.4 degrees celsius, heart rate 64 beats per minute, respiratory rate 18 breaths per minute, blood pressure 123/74 millimeters of mercury, and pulse oximetry 98%. Initial labs were significant for iron deficiency anemia with hemoglobin 7.4 g/dL (normal range 12.0-16.0 g/dL), hematocrit 24.2% (normal range 36.0% to 48.0%), iron 29 ug/dL (normal range 50-170 ug/dL), total iron binding capacity 247 ug/dL (normal range 250-450 ug/dL), and iron saturation 11% (normal range 15% to 50%). Acute kidney injury (AKI) with a creatinine of 2.76 mg/dL (normal range 0.55-1.30 mg/dL) was detected on her basic metabolic panel. Serum glucose on arrival was 160 mg/dL (normal range 74-106 mg/dL). Her coagulation profile including international normalized ratio (INR), prothrombin time (PT), and activated partial thromboplastin time (aPTT) were within normal limits. Erythrocyte sedimentation rate and c-reactive protein were not collected during this encounter. Non-contrast head CT (Figure 1) demonstrated a small hyper-dense focus at the left centrum semiovale, suggestive of a small hyper-dense mass or subacute hemorrhage. 

The patient was admitted to the inpatient medicine service. She was continued on home ART with a recent CD4 count of 284, and the viral load was undetectable. The initial presentation was concerning for a postictal state or transient ischemic attack. She was placed on seizure precautions, neuro checks, and started on levetiracetam. The EEG did not show any epileptic foci. Neurology recommended lumbar puncture and MRI/magnetic resonance angiography (MRA). Due to her history of HIV, recent shingles infection, and small hemorrhagic stroke, there was high suspicion for VZV vasculopathy and she was started on IV acyclovir. Her AKI was treated with IV fluids which were continued for renal protection while on acyclovir. She received 1 unit packed red blood cells, IV iron supplementation, and oral iron supplementation on hospital day (HD) 1 for hemoglobin <7, which improved her anemia while hemoglobin and hematocrit remained stable throughout her hospital stay. 

**Figure 2:**
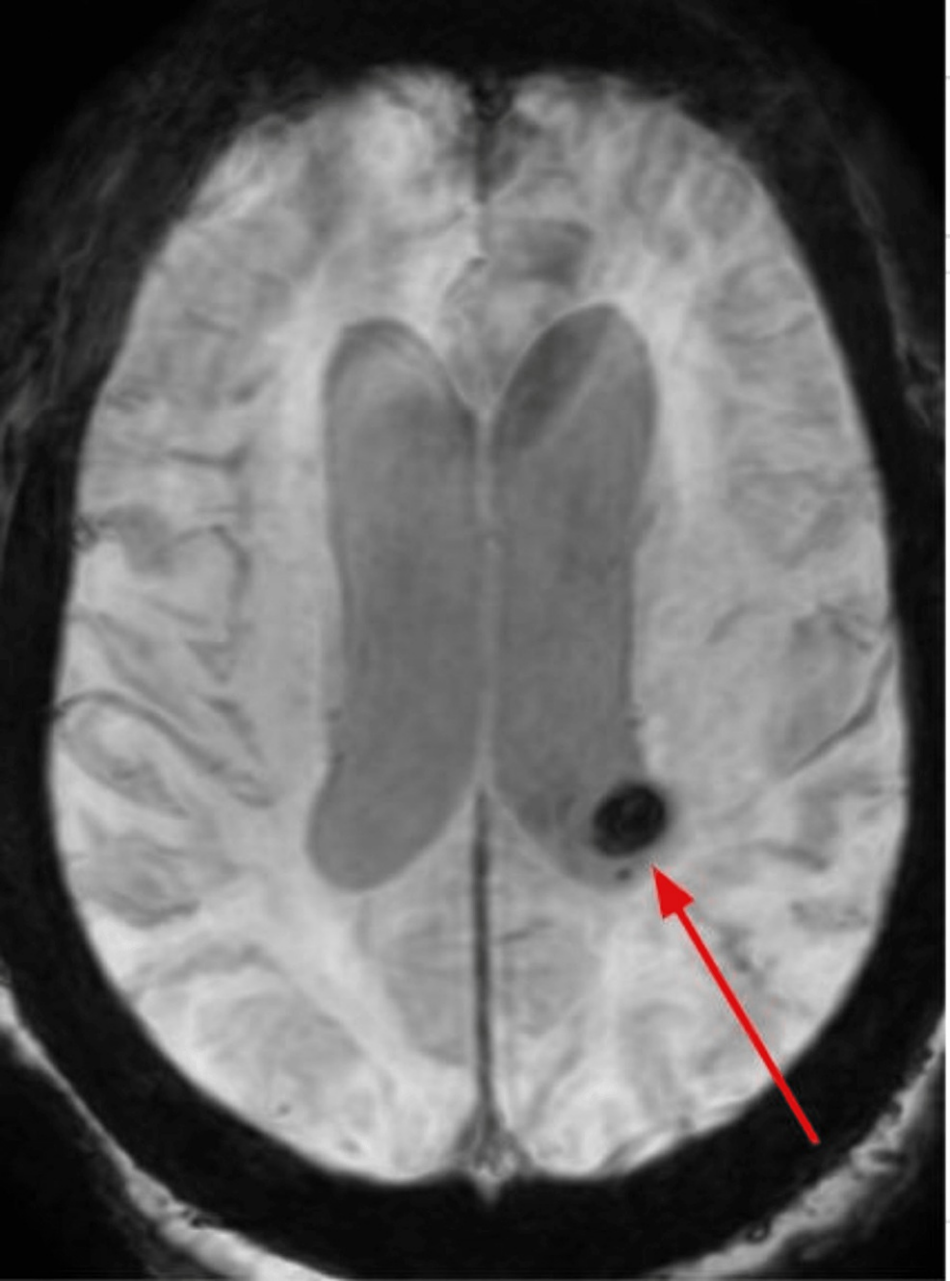
MRI Brain FLAIR showing white matter adjacent to the left lateral ventricle (0.5 cm). These findings are most compatible with a focus of subacute hemorrhage. FLAIR: Fluid-attenuated inversion recovery

**Figure 3:**
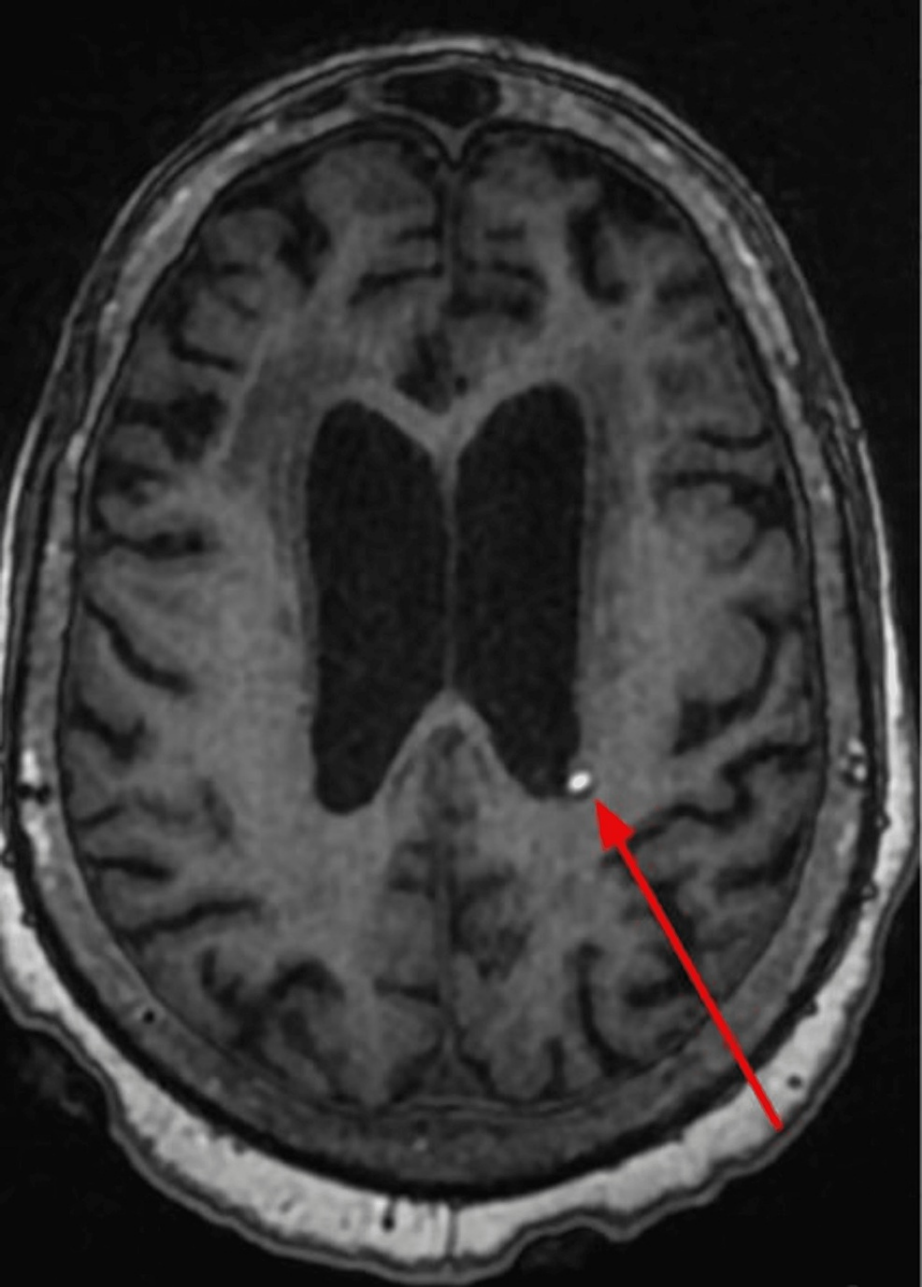
MRI brain T1-weighted image showing increased T1 signal in the white matter adjacent to the left lateral ventricle (0.5 cm). These findings are most compatible with a focus of subacute hemorrhage.

Cerebral spinal fluid findings were consistent with viral etiology with glucose 44, WBC 89 (97% mononuclear), and protein 72. Clinically, her presentation was not consistent with meningitis/encephalitis. The MRI/MRA of the head and neck demonstrated a left periventricular lesion suggestive of a small focus of subacute hemorrhage (Figures 2, 3). The MRA of the head and neck did not show evidence of small or large vessel vasculitis consistent with VZV vasculopathy. Further CSF analysis on HD 7 was not consistent with infection: negative herpes simplex virus (HSV) 1/2, HIV, venereal disease research laboratory (VDRL) test; and VZV DNA by polymerase chain reaction (PCR) was negative but was VZV antibody (Ab) positive. Other diagnoses on the differential included underlying lesion versus hypertensive hemorrhage. However, the patient was not significantly hypertensive while hospitalized or on presentation. She was placed on 14 days of acyclovir with five days of 1 mg/kg prednisone without a taper. She was subsequently discharged from the hospital and transitioned to outpatient care.

## Discussion

Clinical manifestations of VZV vasculopathy can vary widely due to infection of both large and small arteries. When it affects the CNS, it can result in cerebral ischemia or hemorrhage. The classical presentation is ophthalmic-distribution zoster followed by acute contralateral hemiplegia, but it also can occur with headache, mental status change, focal weakness or sensory loss, aphasia, ataxia, hemianopia, or vision loss. Ophthalmic distribution typically leads to unifocal vasculopathy, especially in children and the elderly, while multifocal vasculopathy most commonly occurs in immunocompromised individuals. Some may also present with encephalitis followed by focal deficits. Up to two-thirds of these patients have a history of either zoster infection or varicella rash. The time from rash to neurologic symptoms is sometimes simultaneous but averages about four months apart [3]. Based on a literature review of other published case reports and case series [3], CSF VZV antibodies were positive in the majority of cases. Around 63% of patients present with rash, 67% with pleocytosis, and 97% with imaging abnormalities. Around 30% may have CSF VZV DNA positivity and 93% with positive VZV antibodies. As in the case of our patient, the presence of a rash on presentation is not necessary for diagnosis, but imaging abnormality is suggestive of the disorder. Between 30% to 40% of patients have CNS manifestations without skin involvement [4]. This case is consistent with prior publications where ⅓ of cases have an absent rash and VZV DNA present in the CSF. They presented with vasculopathies ranging anywhere from infarct to cerebral hemorrhage [3,5]. 

Intracerebral VZV vasculopathy occurs with VZV infection of intra and extracranial arteries. After infection, these vessels undergo pathological changes ranging from thrombosis, necrosis, dissection, or aneurysm formation. It has been described as having similar histological changes to giant cell arteritis and granulomatous aortitis. It tends to affect both large and small arteries in the cerebral vasculature. Patients with symptoms consistent with stroke are typically imaged according to local stroke protocol. Brain imaging usually shows ischemic or hemorrhagic infarction. These lesions tend to be bland and multifocal, but single lesions are also reported. An MRI shows lesions in the superficial and deep-seated in both gray and white matter, especially at the gray-white matter junction. After VZV infection there is an increased stroke risk of 2% to 9% in the first year after infection, regardless of the immunological status [6].

A VZV vasculopathy diagnosis involves CSF testing. The CSF analysis usually has a modest pleocytosis (<100 cells/microL) with mononuclear cells. Protein is elevated with normal glucose and oligoclonal bands. Detection of VZV DNA and/or the presence of anti-VZV IgG can help establish the diagnosis. The sensitivity of CSF VZV IgG antibody has been found to be higher than VZV DNA detection by PCR (93% vs 30%) [7]. The absence of VZV DNA in the CSF does not exclude VZV vasculopathy. The VZV DNA is usually detected in the CSF within the first seven days of symptoms but can persist up to 50 days while the CSF VZV IgG antibody is usually detected seven days after symptoms onset [8,9]. 

The first-line treatment for VZV vasculopathy is intravenous acyclovir 10 mg/kg to 15 mg/kg every eight hours for seven to 14 days. [8] In VZV CNS infections, the intravenous formulation should be used due to its very poor oral bioavailability (15% to 30%). The use of steroids (a short course of oral prednisone 1 mg/kg for five to seven days) as an adjunctive treatment is controversial. A previous study showed that 66% of patients treated with acyclovir alone improved or stabilized symptoms compared to 75% of patients with adjunctive steroids. In the general population, the case-fatality rate of VZV vasculopathy without treatment is 25% [3].

## Conclusions

This case highlights the most important clinical aspects regarding clinical presentation, diagnosis, treatment, and complications of VZV vasculopathy in HIV patients. Varicella-zoster virus vasculopathy should be considered in patients with clinical presentation of stroke in the setting of HIV and recent VZV ophthalmopathy. This should be on the differential even in patients with adequate CD4 count, undetectable HIV DNA, and good highly active antiretroviral therapy (HAART) medication compliance. The use of brain imaging and CSF studies are key in the diagnosis. The treatment for this condition is acyclovir administration with or without high-dose steroids and has a high mortality rate if it goes untreated.
